# Protective Effect of *Lonicera japonica* on PM_2.5_-Induced Pulmonary Damage in BALB/c Mice via the TGF-β and NF-κB Pathway

**DOI:** 10.3390/antiox12040968

**Published:** 2023-04-20

**Authors:** Hyo Lim Lee, Jong Min Kim, Min Ji Go, Tae Yoon Kim, Seung Gyum Joo, Ju Hui Kim, Han Su Lee, Hyun-Jin Kim, Ho Jin Heo

**Affiliations:** Division of Applied Life Science (BK21), Institute of Agriculture and Life Science, Gyeongsang National University, Jinju 52828, Republic of Korea; gyfla059@gnu.ac.kr (H.L.L.); myrock201@gnu.ac.kr (J.M.K.); alswl9245@gnu.ac.kr (M.J.G.); s716g@naver.com (S.G.J.); zkfkapflove@nate.com (J.H.K.); ns3005@naver.com (H.S.L.); hyunjkim@gnu.ac.kr (H.-J.K.)

**Keywords:** *Lonicera japonica*, PM_2.5_, pulmonary disease, inflammation, fibrosis

## Abstract

This study aimed to assess the protective effect of an extract of *Lonicera japonica* against particulate-matter (PM)_2.5_-induced pulmonary inflammation and fibrosis. The compounds with physiological activity were identified as shanzhiside, secologanoside, loganic acid, chlorogenic acid, secologanic acid, secoxyloganin, quercetin pentoside, and dicaffeoyl quinic acids (DCQA), including 3,4-DCQA, 3,5-DCQA, 4,5-DCQA, and 1,4-DCQA using ultra-performance liquid chromatography–quadrupole time-of-flight mass spectrometry (UPLC-Q-TOF/MS^E^). The extract of *Lonicera japonica* reduced cell death, reactive oxygen species (ROS) production, and inflammation in A549 cells. The extract of *Lonicera japonica* decreased serum T cells, including CD4^+^ T cells, CD8^+^ T cells, and total T helper 2 (Th2) cells, and immunoglobulins, including immunoglobulin G (IgG) and immunoglobulin E (IgE), in PM_2.5_-induced BALB/c mice. The extract of *Lonicera japonica* protected the pulmonary antioxidant system by regulating superoxide dismutase (SOD) activity, reduced glutathione (GSH) contents, and malondialdehyde (MDA) levels. In addition, it ameliorated mitochondrial function by regulating the production of ROS, mitochondrial membrane potential (MMP), and ATP contents. Moreover, the extract of *Lonicera japonica* exhibited a protective activity of apoptosis, fibrosis, and matrix metalloproteinases (MMPs) via TGF-β and NF-κB signaling pathways in lung tissues. This study suggests that the extract of *Lonicera japonica* might be a potential material to improve PM_2.5_-induced pulmonary inflammation, apoptosis, and fibrosis.

## 1. Introduction

Particulate matter (PM) in the air can penetrate the respiratory system and cause various adverse health effects [1]. When PM enters the body, it triggers an inflammatory response and cytotoxicity in lung tissue [2]. Inhaling PM causes the release of cytokines and chemokines, which are signaling molecules that can cause the activation of immune cells, such as macrophages and T cells [3]. This activation induces the production of reactive oxygen species (ROS) and reactive nitrogen species (RNS), which can cause oxidative stress and damage to lung tissue [4]. PM also leads to the production of proinflammatory mediators, such as interleukins and tumor necrosis factor-α (TNF-α), which can exacerbate the inflammatory response [5]. This response is related to bronchial constriction, leading to breathing difficulties, and causes the accumulation of fluid in the lungs, leading to decreased oxygen exchange [4]. In addition, PM exposure is related to long-term health effects, such as chronic obstructive pulmonary disease (COPD), lung cancer, cardiovascular diseases, and premature death [6]. In particular, long-term exposure to PM induces oxidative stress, leading to damage of the lung tissue and increased risk of lung diseases [7]. The oxidative stress continuously causes systemic inflammation, leading to the development of cardiovascular diseases, such as coronary heart disease and stroke [8]. To reduce the health effects of PM exposure, it is important to decrease the source of PM pollution and take measures to reduce individual exposure to PM [9]. However, since inhalation of PM cannot be completely prevented, it is very important to consume natural materials that can eliminate the stress caused by PM in advance.

*Lonicera japonica* is widely cultivated as an ornamental plant, and it is a native species in eastern Asia, including Japan, China, and Korea [10]. It has been reported to have antioxidant properties, which may help protect the body against cellular damage caused by free radicals and oxidative stress [11]. Extracts of *Lonicera japonica* can inhibit the growth of bacteria, fungi, and viruses [12]. In addition, *Lonicera japonica* showed protective effects against hepatic damage, neuronal death, and ulcerative colitis [13,14,15].

These physiological activities suggest that *Lonicera japonica* is a promising candidate for further study as a source of natural materials for human health. Especially, *Lonicera japonica* showed the protective effect of pulmonary cytotoxicity via regulation of inflammatory reaction in lipopolysaccharides (LPSs) and an ovalbumin-induced mice model [16,17,18]. However, there are few studies related to the ameliorating effect of *Lonicera japonica* on pulmonary damage and inflammatory response caused by PM_2.5_ exposure. Although some studies, such as the improvement activity against intestinal dysfunction [19] and acute pulmonary toxicity [20] induced by PM_2.5_, have been reported, these studies on *Lonicera japonica* complex extracts are studies related to acute toxicity due to short-term exposure, and a demonstration of the amelioration activity of toxicity by long-term exposure is limited. Therefore, in this study, the protective effect of *Lonicera japonica* extract was evaluated against chronically PM_2.5_-induced BALB/c mice.

## 2. Materials and Methods

### 2.1. Chemicals

Roswell Park Memorial Institute (RPMI) 1640, fetal bovine serum (FBS), penicillin, streptomycin, 3-(4,5-Dimethylthiazol-2-yl)-2,5-diphenyltetrazolium bromide (MTT), 2′,7′-dichlorofluorescein diacetate (DCF-DA), phenylmethane sulfonylfluoride, metaphosphoric acid, o-phthaldialdehyde, o-phosphoric acid, thiobarbituric acid (TBA), trichloroacetic acid (TCA), sucrose, bovine serum albumin (BSA), pyruvic acid, malic acid, mannitol, HEPES sodium salt, egtazic acid (EGTA), digitonin, 5,5,6,6-tetrachloro-1,1,3,3-tetraethylbenzimidazolylcarbocyanine iodide (JC-1), and solvents were purchased from Sigma-Aldrich Chemical Corp (St. Louis, MO, USA). A superoxide dismutase (SOD) determination kit was purchased from Dojindo Molecular Technologies (Kumamoto, Japan). An ATP assay kit was purchased from Promega Corp. (Madison, WI, USA). PM_2.5_ (mean diameter: 1.06 μm) was purchased from Power Technology Inc (Arizona Test Dust, Arden Hills, MN, USA).

### 2.2. Sample Preparation

*Lonicera japonica* used in this experiment was obtained from Bigsomebio (Jinju, Republic of Korea) in September 2022. A dried sample was extracted with 30% ethanol at 50 °C for 2 h. The extracted sample was filtered with filter paper (Advantec No. 2 330 mm, Advantec Co., Ltd., Tokyo, Japan) and concentrated using a vacuum rotary evaporator (N-N series, Eyela Co., Tokyo, Japan). Extracts of *Lonicera japonica* were dried using a vacuum tray dryer (FDU-8612, Operon, Gimpo, Republic of Korea) and stored at −20 °C before use in each experiment.

### 2.3. Physiological Compound Analysis

In order to identify the compounds with physiological activity, the extracts of *Lonicera japonica* were dissolved in 50% methanol and were then analyzed using ultra-performance liquid chromatography–quadrupole time-of-flight mass spectrometry (UPLC-IMS-Q-TOF/MS^E^, Vion, Waters Corp., Milford, MA, USA) with a BEH C_18_ column (100 × 2.1 mm, 1.7 μm; Waters Corp.). The mobile phases consisted of solvent A (0.1% formic acid in distilled water) and solvent B (0.1% formic acid in acetonitrile) during analysis, and gradient conditions were as follows: 0.1% B at 0–1 min, 0–100% B at 1–8 min, 100% B at 8–9 min, 100–0.1% B at 9–9.5 min, 0.1% B at 9.5–12 min. The conditions used for the electrospray ionization (ESI) source were as follows: ramp collision energy, 20–45 V; oven temperature, 40 °C; capillary voltage, 3 kV; pressure of nebulizer, 40 psi; fragmentor, 175 V; cone voltage, 40 V; mass range, 50–1500 *m*/*z*. The UPLC-Q-TOF/MS^E^ system was analyzed using data analysis software (Waters Masslynx™ 4.1 version, Waters Corp.).

### 2.4. Evaluation of Pulmonary Protective Effect

#### 2.4.1. Cell Culture and Treatment

A549 cells isolated from lung tissue cell lines were acquired from the Korean Cell Line Bank (Seoul, Republic of Korea) and incubated in RPMI 1640 medium with 10% FBS, 50 units/mL penicillin, and 100 μg/mL streptomycin in the conditions of 5% CO_2_ at 37 °C.

#### 2.4.2. Cell Viability

In order to evaluate the cellular viability, A549 cells (10^4^ cells/well) were treated with the extracts of *Lonicera japonica*. After incubating for 3 h, the cells were treated with 100 μM PM_2.5_. After 24 h, 5 mg/mL of MTT solution were treated into each well for 3 h. The MTT formazan contents were measured using a microplate reader (Epoch 2, BioTek Instruments, Inc., Winooski, VT, USA) at a determination wavelength of 570 nm and a reference wavelength of 690 nm [21].

#### 2.4.3. Reactive Oxygen Species (ROS) Contents

In order to evaluate the inhibitory effect of intracellular ROS, A549 cells (10^4^ cells/well) were treated with the extracts of *Lonicera japonica*. After incubating for 3 h, the cells were treated with 100 μM PM_2.5_. After 24 h, 10 μM DCF-DA dissolved in phosphate-buffered saline (PBS) were treated into each well for 3 h. The ROS production was measured using a fluorescence microplate reader (Infinite 200, Tecan Co., San Jose, CA, USA) at 485 nm excitation and with 530 nm emission filters [22].

### 2.5. Animal Experimental Design

BALB/c mice (8 weeks old, male, *n* = 13; five for ex vivo tests; five for mitochondrial tests; three for Western blot analysis) were purchased from Samtako (Osan, Republic of Korea). The experimental animals were randomly divided into five per cage and administrated in standard laboratory conditions (12 h light/dark cycle, 55% humidity, and 22 ± 2 °C). Experimental groups were divided into seven groups (1. a sham control (Sham) group without chamber exposure; 2. a clean-air-exposed normal control (NC) group; 3. a clean-air-exposed control group treated with the extracts of *Lonicera japonica* (100 mg/kg of body weight; NS); 4. a PM_2.5_-exposed (negative control group; PM) group; 5–7. PM_2.5_-exposed groups treated with the extracts of *Lonicera japonica* (20, 50, and 100 mg/kg of body weight; EL20, EL50, and EL100, respectively). The extracts of *Lonicera japonica* were dissolved in filtered drinking water and fed using a stomach tube, as an oral zonde needle, once a day for 12 weeks. PM_2.5_ exposure was applied as a 500 μg/m^3^ concentration using a whole-body-exposure chamber for 5 h/day for 12 weeks according to World Health Organization (WHO) air quality guidelines and a previous study. All animal procedures were conducted according to the Institutional Animal Care and Use Committee of Gyeongsang National University (certificate: GNU-220831-M0098, approved on 31 August 2022) and performed in accordance with the Policy of the Ethical Committee of the Ministry of Health and Welfare, Republic of Korea.

### 2.6. Determination of T Cells by Flow Cytometry

In order to measure T cell levels, the collected blood was stained with APC-Cy7-conjugated CD3^+^, PE-Cy7-conjugated CD4^+^, and PerCp-Cy5.5-conjugated CD8^+^ at room temperature for 15 min in the dark in a separate reaction. Incubated blood was reacted with lysing solution (349202, BD Biosciences, Franklin Lakes, NJ, USA) at room temperature for 15 min in the dark. Reacted blood was centrifuged at 1200× *g* for 5 min at 4 °C, and the pellet was washed using stain buffer (#554657, BD Biosciences). The washed pellet was fixed and permeabilized using a Fixation/Permeabilization Solution Kit (#554715, BD Biosciences) for 20 min at 4 °C. The fixed and permeabilized pellet was reacted and stained with PE-conjugated IL-4^+^ mAb to stain intracellular cytokines. The sample was centrifuged at 1200× *g* for 5 min at 4 °C using stain buffer for washing. The sample was analyzed using a BD FACS Canto Ⅱ flow cytometer (BD Biosciences).

### 2.7. Serum Immunoglobulins (Ig) by ELISA

In order to measure serum Ig levels, the collected blood was centrifuged at 10,000× *g* for 15 min at 4 °C. This supernatant was measured for Ig levels using a commercial IgG kit (Abbkine, Wuhan, China) and an IgE kit (Abcam, Cambridge, UK). 

### 2.8. Pulmonary Antioxidant System

#### 2.8.1. SOD Contents

In order to measure SOD contents, homogenized pulmonary tissue in PBS was centrifuged at 400× *g* for 10 min at 4 °C. The pellets were treated with 1 × cell extraction buffer containing 10% SOD buffer, 0.4% (*v*/*v*) Triton X-100 and 200 μM phenylmethane sulfonylfluoride, and centrifuged at 10,000× *g* for 10 min at 4 °C. The supernatants were measured for the SOD contents using a commercial SOD kit (Dojindo Molecular Technologies).

#### 2.8.2. Reduced Glutathione (GSH) Contents

In order to measure reduced GSH contents, the homogenized pulmonary tissue in phosphate buffer (pH 6.0) was centrifuged at 10,000× *g* for 15 min at 4 °C. This supernatant was reacted with 5% metaphosphoric acid and centrifuged at 2000× *g*. The supernatant was reacted with 0.26 M of tris-HCl (pH 7.8), 0.65 N of NaOH, and 1 mg/mL of o-phthaldialdehyde at room temperature. After 15 min, the reacted fluorescence was measured using a fluorescence microplate reader (Infinite 200, Tecan Co., Männedorf, Switzerland) at 320 nm (excitation) and 420 nm (emission) [23].

#### 2.8.3. Malondialdehyde (MDA) Contents

In order to measure MDA contents, the homogenized tissues in PBS were centrifuged at 5000 rpm for 10 min at 4 °C. The supernatants were reacted with 1% o-phosphoric acid and 0.67% thiobarbituric acid in a 95 °C for 1 h. The reactants were centrifuged at 600× *g* for 10 min, and the supernatants were measured at 532 nm (UV-1800, Shimadzu, Tokyo, Japan) [23].

### 2.9. Pulmonary Mitochondrial Function

#### 2.9.1. Mitochondrial Isolation

Pulmonary tissues homogenized in a mitochondria isolation (MI) buffer (215 mM mannitol, 75 mM sucrose, 0.1% BSA, 20 mM HEPES sodium salt, and 1 mM EGTA, pH 7.2) were centrifuged at 1300× *g* for 5 min at 4 °C. The supernatant was centrifuged at 13,000× *g* for 10 min at 4 °C. The obtained pellets were reacted with an MI buffer containing 1 mM EGTA and 0.1% digitonin, and recentrifuged at 13,000× *g* for 15 min at 4 °C. The obtained pellets were reacted with MI buffer and centrifuged at 10,000× *g* for 10 min at 4 °C. The mitochondrial activities were assessed using the finally obtained pellets.

#### 2.9.2. Mitochondrial ROS Contents

In order to measure mitochondrial ROS production, the obtained pellets were reacted with KCl-based respiration buffer (125 mM potassium chloride, 2 mM potassium phosphate monobasic, 2.5 mM malate, 20 mM HEPES, 1 mM magnesium chloride, 5 mM pyruvate, and 500 μM EGTA, pH 7.0) and 10 μM DCF-DA for 20 min. ROS production was measured using a fluorescence microplate reader (Infinite 200, Tecan Co., San Jose, CA, USA) at an excitation wave of 485 nm and an emission wave of 535 nm [24].

#### 2.9.3. Mitochondrial Membrane Potential (MMP)

In order to measure MMP, the obtained pellets were reacted with MI buffer containing 5 mM pyruvate and 5 mM malate. The reactants were reacted with 1 μM JC-1 in the dark for 20 min. The MMP levels were measured using a fluorescence microplate reader (Infinite 200, Tecan Co., San Jose, CA, USA) at an excitation wave of 530 nm and an emission wave of 590 nm [24]. 

#### 2.9.4. ATP Contents

In order to measure mitochondrial ATP content, the mitochondrial extracts were centrifuged at 13,000× *g* for 10 min at 4 °C. The pellet was reacted with 1% TCA on ice for 10 min. The reactants were mixed with 25 mM tris-acetate buffer (pH 7.7) and centrifuged at 10,000× *g* for 15 min at 4 °C. The supernatants were used for measuring the mitochondrial ATP content using an ATP assay kit (Promega Corp.) using a luminometer (GloMax-Multi Detection System, Promega Corp., Madison, WI, USA).

### 2.10. Western Blot

The pulmonary tissues were homogenized in lysis buffer (GeneAll Biotechnology, Seoul, Republic of Korea) with a 1% protease inhibitor (Quartett, Berlin, Germany) for 10 min. The obtained supernatants centrifuged at 13,000× *g* for 10 min at 4 °C were separated by SDS-PAGE gel and electro-transferred to a polyvinylidene difluoride membrane (Milipore, Billerica, MA, USA). After blocking with skim milk at room temperature for 1 h, the membrane-combined proteins were reacted overnight in primary antibodies at 4 °C, and secondary antibodies for 1 h at room temperature. The luminescence of the immune complexes was detected using a Western blot image analyzer (iBright Imager, Thermo-Fisher Scientific, Waltham, MA, USA). To calculate the density value of each factor, the loading control was used as β-actin. Antibody details are presented in Table 1.

### 2.11. Statistical Anaylsis

All data were presented as mean ± standard deviation (SD). Significant differences between each group were analyzed by one-way analysis and determined using Duncan’s new multiple range test (*p* < 0.05) of SAS ver. 9.4 (SAS Institute Inc., Cary, NC, USA), and different small letters represent statistical differences. The in vitro cell studies, Western blot experiments, and serum analysis were repeated 3 times, and tissue antioxidant system and mitochondrial experiments were repeated 5 times.

## 3. Results

### 3.1. Physiological Compound of Lonicera japonica

**Figure 1:**
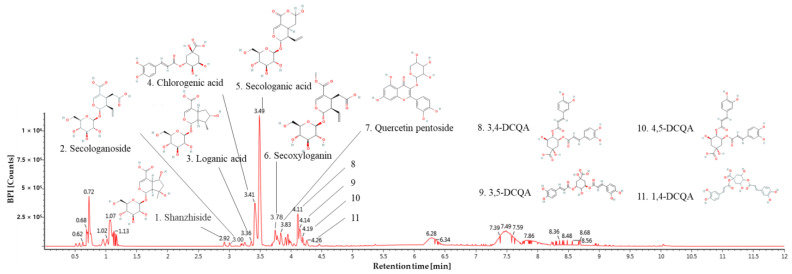
UPLC Q–TOF/MS^E^ chromatography in negative ion mode of *Lonicera japonica*.

The physiological compounds of the extracts of *Lonicera japonica* were qualitatively identified using UPLC-Q-TOF/MS^E^ (Figure 1 and Table 2). The MS^E^ spectra were analyzed in negative ion mode as compound **1**: 391 *m*/*z* (retention time (RT): 2.92 min); compound **2**: 389 *m*/*z* (RT: 3.00 min); compound **3**: 375 *m*/*z* (RT: 3.36 min); compound **4**: 353 *m*/*z* (RT: 3.41 min); compound **5**: 373 *m*/*z* (RT: 3.49 min), compound **6**: 403 *m*/*z* (RT: 3.78 min), compound **7**: 433 *m*/*z* (RT: 3.83 min), compound **8**: 515 *m*/*z* (RT: 4.11 min), compound **9**: 515 *m*/*z* (RT: 4.14 min), compound **10**: 515 *m*/*z* (RT: 4.19 min), and compound **11**: 515 *m*/*z* (RT: 4.26 min). These compounds were tentatively identified as shanzhiside (compound **1**), secologanoside (compound **2**), loganic acid (compound **3**), chlorogenic acid (compound **4**), secologanic acid (compound **5**), secoxyloganin (compound **6**), quercetin pentoside (compound **7**), 3,4-O-dicaffeoylquinic acid (DCQA) (compound **8**), 3,5-O-DCQA (compound **9**), 4,5-O-DCQA (compound **10**), and 1,4-O-DCQA (compound **11**) using Waters MassLynx™ (Waters Corp.) library software and previous studies [25,26,27].

### 3.2. Protective Effect of A549 Cells

#### 3.2.1. Cell Viability

**Figure 2:**
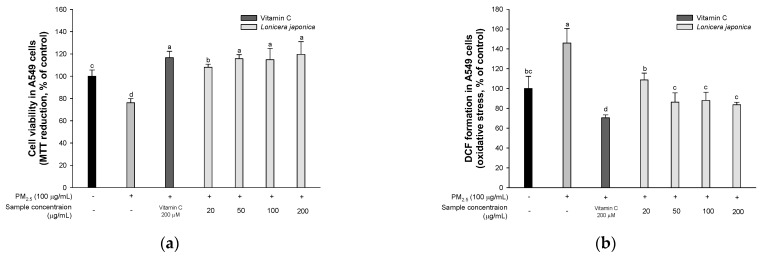

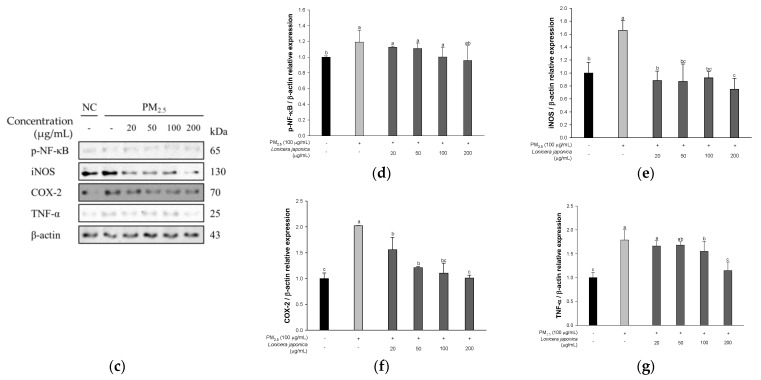
Pulmonary protective effect of extract of *Lonicera japonica* on PM_2.5_-induced cytotoxicity. (**a**) Cell viability; (**b**) Reactive oxygen species (ROS) Contents; (**c**) Western blot images; Protein expression levels of p-NF-κB (**d**), iNOS (**e**), COX-2 (**f**), and TNF-α (**g**). Results shown are mean ± SD (*n* = 3). Data were statistically represented at *p* < 0.05, and different lowercase letters indicate statistical significance.

To evaluate the pulmonary protective effect of the extracts of *Lonicera japonica*, cell viability was measured in A549 cells (Figure 2a). The cell viability of the PM_2.5_-induced group (76.22%) was reduced compared to the normal control group (100%). However, the vitamin-C- and sample-treated groups increased the cell viability (vitamin C, 116.67%; 100 μg/mL, 114.88%; 200 μg/mL, 119.63%, respectively) compared to the H_2_O_2_-induced groups.

#### 3.2.2. ROS Production

To evaluate the pulmonary protective effect of the extracts of *Lonicera japonica*, ROS production was measured in A549 cells (Figure 2b). The ROS production of the PM_2.5_-induced group (145.90%) was increased compared to the normal control group (100%). However, the vitamin-C- and sample-treated groups suppressed the ROS production (vitamin C, 70.47%; 100 μg/mL, 87.97%; 200 μg/mL, 83.62%, respectively) compared to the H_2_O_2_-induced groups.

#### 3.2.3. Protein Expression of Inflammation in A549 Cells

The protein expressions related to the inflammatory pathway in A549 cells are presented in Figure 2c–g. The p-NF-κB (119.13%), iNOS (165.64%), COX-2 (202.18%), and TNF-α (178.87%) expression levels in the PM_2.5_-treated group were significantly upregulated compared to the NC group (100%). However, the sample-treated groups statistically downregulated the p-NF-κB (100 μg/mL, 100.04%; 200 μg/mL, 95.71%), iNOS (100 μg/mL, 92.4%; 200 μg/mL, 74.87%), COX-2 (100 μg/mL, 110.57%; 200 μg/mL, 100.98%), and TNF-α (100 μg/mL, 155.10%; 200 μg/mL, 115.01%) expression levels compared to the PM_2.5_-treated group. 

### 3.3. Serum Inflammatory Cytokines

#### 3.3.1. T Cells

**Figure 3:**
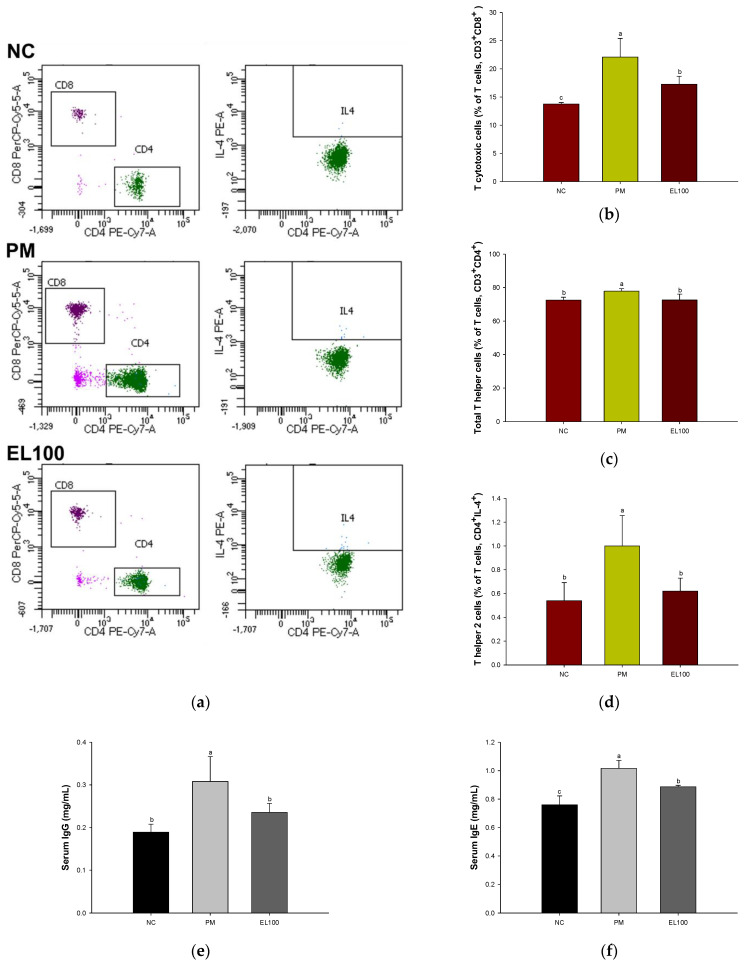
Protective effect of extract of *Lonicera japonica* on PM_2.5_-induced inflammatory response. (**a**) Flow cytometer analysis; (**b**) T cytotoxicity cells; (**c**) Total T helper cells; (**d**) T helper 2 cells; (**e**) IgG contents; (**f**) IgE contents. Results shown are mean ± SD (*n* = 3). Data were statistically represented at *p* < 0.05, and different lowercase letters indicate statistical significance.

To evaluate the anti-inflammatory effect of the extracts of *Lonicera japonica*, T cell levels were measured in serum (Figure 3a–d). The T cytotoxicity cell (CD3^+^CD8^+^), total T helper cell (CD3^+^DC4^+^), and T helper 2 cell (CD4^+^IL-4^+^) levels of the PM group (22.10, 77.88, and 0.54% of T cells) was increased compared to the NC group (13.76, 72.50, and 1.00% of T cells). However, the EL100 group suppressed the T cytotoxicity cell (CD3^+^CD8^+^), T helper cell (CD3^+^DC4^+^), and T helper 2 cell (CD4^+^IL-4^+^) (17.28, 72.58, and 0.62% of T cells) levels compared to the PM group.

#### 3.3.2. Immunoglobulins

To evaluate the anti-inflammatory effect of the extracts of *Lonicera japonica*, IgG and IgE levels were measured in serum (Figure 3e,f). The IgG and IgE levels of the PM group (0.31 and 1.02 mg/mL) were increased compared to the NC group (0.19 and 0.76 mg/mL). However, the EL100 group suppressed the IgG and IgE levels (0.24 and 0.89 mg/mL) compared to the PM group.

### 3.4. Antioxidant System in Lung Tissue

#### 3.4.1. SOD Activities

**Figure 4:**
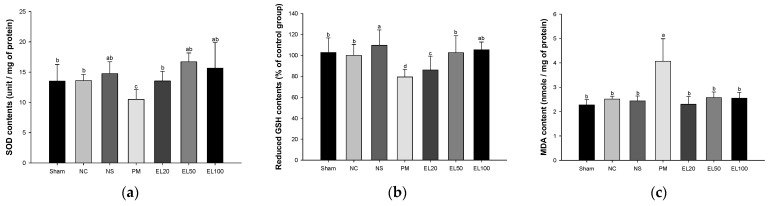
Protective effect of extract of *Lonicera japonica* on PM_2.5_-induced biochemical changes related to the antioxidant system. (**a**) Superoxide dismutase (SOD) contents; (**b**) Reduced glutathione (GSH) contents; (**c**) Malondialdehyde (MDA) levels. Results shown are mean ± SD (*n* = 5). Data were statistically represented at *p* < 0.05, and different lowercase letters indicate statistical significance.

The pulmonary SOD activity is presented in Figure 4a. The SOD activity among the Sham (13.53 unit/mg of protein), NC (13.62 unit/mg of protein), and NS (14.76 unit/mg of protein) groups showed no significant differences. The PM group (20.50 unit/mg of protein) was significantly compared to the NC group. However, the EL groups (EL20, 13.55%; EL50, 16.72%; EL100, 15.66%) were significantly increased compared to the PM group.

#### 3.4.2. Reduced GSH Contents

The pulmonary reduced GSH contents are presented in Figure 4b. Reduced GSH contents among the Sham (102.72%), NC (100.00%), and NS (109.63%) groups showed no significant differences. The PM group (79.38%) was significantly reduced compared to the NC group. However, the EL groups (EL20, 86.07%; EL50, 102.56%; EL100, 105.38%) were significantly increased compared to the PM group.

#### 3.4.3. MDA Levels

The pulmonary MDA contents are presented in Figure 4c. The MDA contents among the Sham (2.28 nmole/mg), NC (2.51 nmole/mg), and NS (2.44 nmole/mg) groups showed no significant differences. The PM group (4.06 nmole/mg) was significantly increased compared to the NC group. However, the EL groups (EL20, 2.30 nmole/mg; EL50, 2.57 nmole/mg; EL100, 2.55 nmole/mg) were significantly attenuated compared to the PM group.

### 3.5. Mitochondrial Function

#### 3.5.1. Mitochondrial ROS Contents

**Figure 5:**
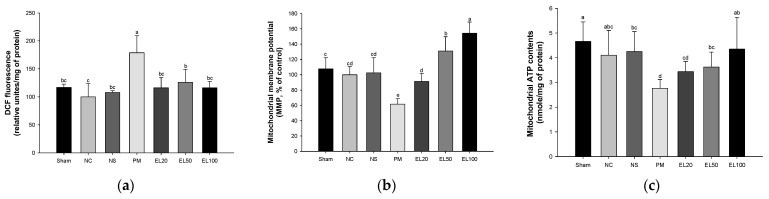
Protective effect of extract of *Lonicera japonica* on PM_2.5_-induced biochemical changes related to the mitochondrial function. (**a**) Mitochondrial reactive oxygen species (ROS) contents; (**b**) Mitochondrial membrane potential (MMP) levels; (**c**) Mitochondrial ATP contents. Results shown are mean ± SD (*n* = 5). Data were statistically represented at *p* < 0.05, and different lowercase letters indicate statistical significance.

The pulmonary mitochondrial ROS contents are presented in Figure 5a. The mitochondrial ROS contents among the Sham (116.88%), NC (100.00%), and NS (107.75%) groups showed no significant differences. The PM group (178.83%) was significantly increased compared to the NC group. However, the EL groups (EL20, 116.09%; EL50, 125.99%; EL100, 116.09%) were significantly decreased compared to the PM group.

#### 3.5.2. Mitochondrial Membrane Potential (MMP)

The pulmonary MMP levels are presented in Figure 5b. The MMP levels among the Sham (107.66%), NC (100.00%), and NS (102.58%) groups showed no significant differences. The PM group (61.53%) was significantly reduced compared to the NC group. However, the EL groups (EL20, 91.40%; EL50, 130.96%; EL100, 154.36%) were significantly increased compared to the PM group.

#### 3.5.3. Mitochondrial ATP Contents

The pulmonary ATP levels are presented in Figure 5c. The ATP contents among the Sham (4.66 nmole/mg of protein), NC (4.10 nmole/mg of protein), and NS (4.45 nmole/mg of protein) groups showed no significant differences. The PM group (2.76 nmole/mg of protein) was significantly reduced compared to the NC group. However, the EL groups (EL20, 3.44 nmole/mg of protein; EL50, 3.63 nmole/mg of protein; EL100, 4.35 nmole/mg of protein) were significantly increased compared to the PM group.

### 3.6. Protein Expression of Inflammatory Pathway

**Figure 6:**
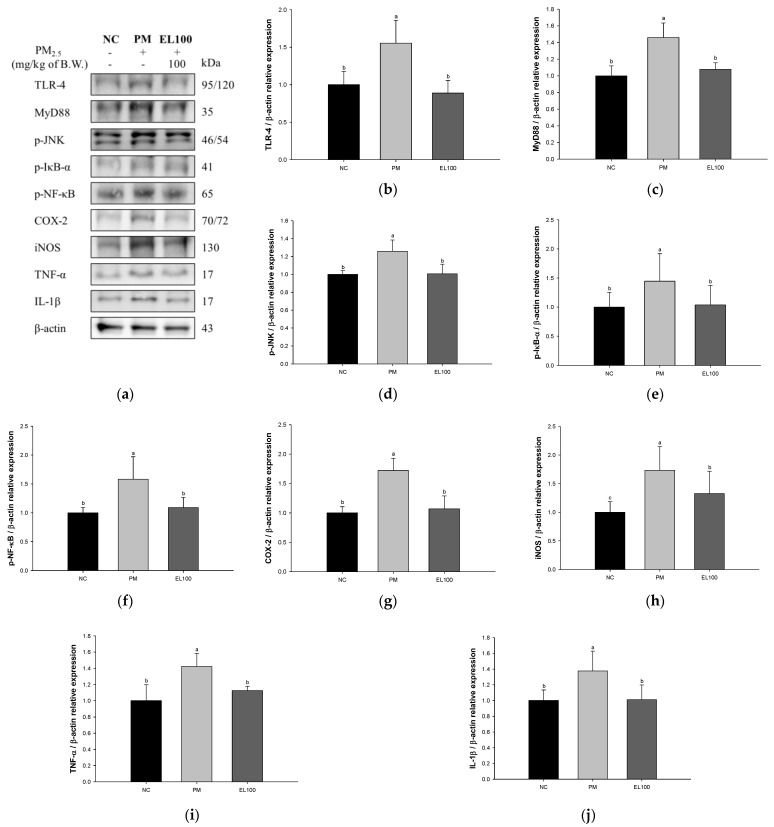
Protective effect of extract of *Lonicera japonica* on PM_2.5_-induced protein expression of Western blot images (**a**). Protein expression levels of TLR-4 (**b**), MyD88 (**c**), p-JNK (**d**), p-NF-κB (**e**), p-NF-κB (**f**), COX-2 (**g**), iNOS (**h**), TNF-α (**i**), and IL-1β (**j**). Results shown are mean ± SD (*n* = 3). Data were statistically represented at *p* < 0.05, and different lowercase letters indicate statistical significance.

The pulmonary protein expressions related to the inflammatory pathway are presented in Figure 6. Toll-like receptor 4 (TLR-4) (155.43%), myeloid differentiation primary response 88 (MyD88) (146.03%), phosphorylated c-Jun N-terminal kinases (p-JNK) (125.79%), phosphorylated nuclear factor of kappa light polypeptide gene enhancer in B-cells inhibitor, alpha (p-IκB-α) (144.54%), phosphorylated nuclear factor kappa-light-chain-enhancer of activated B cells (p-NF-κB) (158.07%), cyclooxygenase-2 (COX-2) (172.01%), inducible nitric oxide synthase (iNOS) (173.43%), TNF-α (142.21%), and interleukin-1β (IL-1β) (137.56%) expression levels in the PM group were significantly upregulated compared to those in the NC group (100%). However, the EL100 group statistically downregulated TLR-4 (88.89%), MyD88 (107.78), p-JNK (100.60%), p-IκB-α (103.78%), p-NF-κB (108.96%), COX-2 (106.96%), iNOS (132.76%), TNF-α (112.41%), and IL-1β (101.10%) expression levels compared to the PM group.

### 3.7. Protein Expression of Apoptosis

**Figure 7:**
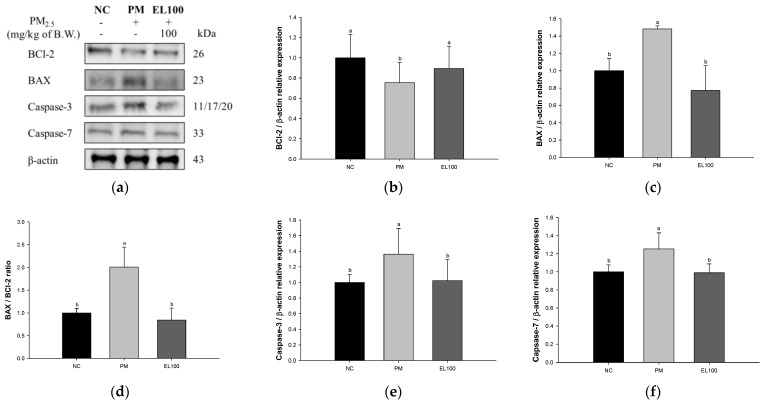
Protective effect of extract of *Lonicera japonica* on PM_2.5_-induced protein expression of Western blot images (**a**). Protein expression levels of BCl-2 (**b**), BAX (**c**), BAX/BCl-2 ratio (**d**), Caspase-3 (**e**), and Caspase-7 (**f**). Results shown are mean ± SD (*n* = 3). Data were statistically represented at *p* < 0.05, and different lowercase letters indicate statistical significance.

The pulmonary protein expressions related to the apoptosis pathway are presented in Figure 7. The expression levels of B-cell lymphoma 2 (BCl-2) (75.37%) in the PM group were significantly downregulated compared to the NC group (100%) (Figure 6b). However, the EL100 group statistically upregulated BCl-2 (89.43%) expression levels compared to the PM group. Bcl-2-associated X protein (BAX) (148.31%), BAX/BCl-2 ratio (200.78%), Caspase-3 (136.23%), and Caspase-7 (125.34%) expression levels in the PM group were significantly upregulated compared to the NC group (100%) (Figure 6c–e). However, the EL100 group statistically downregulated BAX (77.14%), BAX/BCl-2 ratio (84.32%), Caspase-3 (102.34%), and Caspase-7 (98.96%) expression levels compared to the PM group. 

### 3.8. Protein Expression of Pulmonary Fibrosis

**Figure 8:**
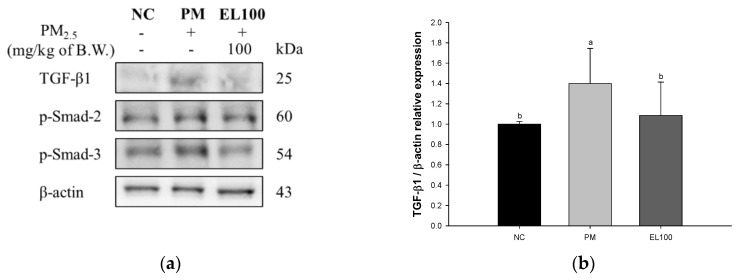

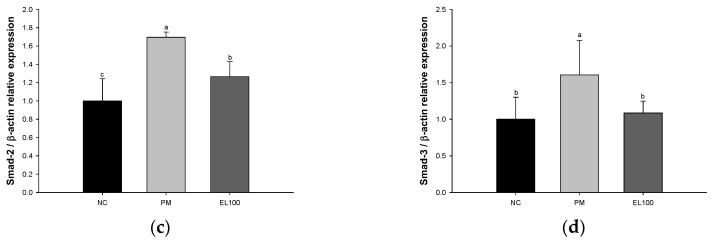
Protective effect of extract of *Lonicera japonica* on PM_2.5_-induced protein expression of Western blot images (**a**). Protein expression levels of TGF-β1 (**b**), p-Smad-2 (**c**), and p-Smad-3 (**d**). Results shown are mean ± SD (*n* = 3). Data were statistically represented at *p* < 0.05, and different lowercase letters indicate statistical significance.

The pulmonary protein expressions related to the apoptosis pathway are presented in Figure 8. Transforming growth factor beta (TGF-β1) (139.93%), phosphorylated small mothers against decapentaplegic (p-Smad)-2 (169.55%), and p-Smad-3 (160.48%) expression levels in the PM group were significantly upregulated compared to the NC group (100%). However, the EL100 group statistically downregulated TGF-β1 (108.49%), p-Smad-2 (126.52%), and p-Smad-3 (108.49%) expression levels compared to the PM group. 

### 3.9. Protein Expression of Matrix Metalloproteinases (MMPs)

**Figure 9:**
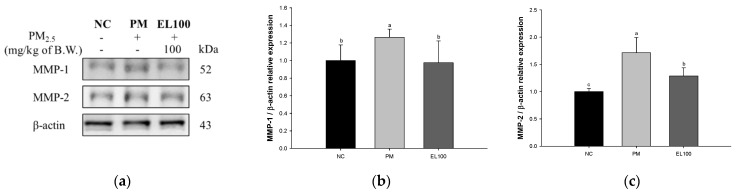
Protective effect of extract of *Lonicera japonica* on PM_2.5_-induced protein expression of Western blot images (**a**). Protein expression levels of MMP-1 (**b**) and MMP-2 (**c**). Results shown are mean ± SD (*n* = 3). Data were statistically represented at *p* < 0.05, and different lowercase letters indicate statistical significance.

The pulmonary protein expressions related to the apoptosis pathway are presented in Figure 9. MMP-1 (126.50%) and MMP-2 (171.39%) expression levels in the PM group were significantly upregulated compared to the NC group (100%). However, the EL100 group statistically downregulated MMP-1 (97.58%) and MMP-2 (128.80%) expression levels compared to the PM group. 

## 4. Discussion

PM_2.5_ is an environmental pollutant that causes various health problems, and exposure to PM_2.5_ causes diseases such as respiratory disease, lung cancer, and fibrosing bronchitis [9]. PM_2.5_ absorbed into the lung tissue induces various inflammatory reactions, and PM_2.5_ and its oxidative properties cause damage to the lung tissue by inducing death and fibrosis of lung cells [3]. The protective effects and specific mechanisms of the extracts of *Lonicera japonica* to inhibit this damage and protect against external stress are not clear. Therefore, this study was conducted to confirm the protective effect of the extracts of *Lonicera japonica* against damage caused by PM_2.5_ inhalation.

When PM_2.5_ reaches the lung tissue, it causes damage to the antioxidant system that protects the lung tissue [28]. The lungs have a structure that is vulnerable to fine dust and is an organ that comes in direct contact with PM_2.5_, and damage to lung tissue causes inflammatory reactions by producing cytokines throughout the body [29]. In particular, various heavy metals contained in PM_2.5_ increase the production of ROS, such as hydroxyl radicals and superoxides, by reducing antioxidant enzymes, including catalase (CAT), SOD, and GSH in lung tissues [30]. The ROS resulting from this process ultimately damage lung cells and lead to impaired lung function. Therefore, to evaluate the ameliorating effect of the extracts of *Lonicera japonica* on antioxidant system damage, SOD activity, reduced GSH levels, and MDA production in pulmonary tissues were assessed, and the extracts significantly protected the pulmonary antioxidant system (Figure 4). Similar to this study, the administration of polysaccharides of *Lonicera japonica* showed protective effects on hepatic antioxidant deficits by increasing the expression levels of CAT, SOD, and GSH in type 2 diabetic SD rats [31]. The ethanol extract of *Lonicera japonica* flower buds showed neuronal protective effects against glutamate-induced hippocampal cytotoxicity by reducing ROS production and increasing the GSH and SOD levels in HT22 cells [32]. The aqueous extract of *Lonicera japonica*, including neochlorogenic acid, chlorogenic acid, cryptochlorogenic acid, secoxyloganin, and secologanic acid, showed increases in SOD, CAT, and GSH/oxidized GSH (GSSG) ratios, and reduction of MDA levels by inhibiting the inflammatory response in gastric mucosal tissues [33]. In particular, chlorogenic acid, one of the physiological compounds of the extract of *Lonicera japonica*, ameliorated lead (Pb)-induced renal damage by regulating SOD and GSH peroxidase (GSH-Px) activities [34]. In addition, quercetin protected against antioxidant losses induced by various toxic ions such as cadmium (Cd), Pb, iron (Fe), and aluminum (Al) [35]. In conclusion, the administration of the extract of *Lonicera japonica* significantly protected against pulmonary antioxidant losses from PM_2.5_-induced stresses. Thus, the extract of *Lonicera japonica* might be a potential material with antioxidant activity for functional foods, complementary medicines, or antioxidants.

It has been reported that oxidative stress and inflammatory responses induced by PM_2.5_ affect the mitochondrial function in respiratory tissues [36]. Mitochondria are cellular organelles that help with energy supply and genetic function through oxidative phosphorylation [37]. Mitochondrial damage induced by particulate matter can lead to an imbalance in ATP production and an increase in ROS production, resulting in changes in mitochondrial morphology [38]. PM_2.5_ causes morphological changes in mitochondria, such as swelling, cristae disorder, vacuolation, and fission [36]. Swollen mitochondria induce mitochondrial membrane dysfunction and damage, and the enzyme activity of mitochondrial Na^+^K^+^-ATPase and Ca^2+^-ATP are reduced [39]. These results suggest that PM_2.5_-induced dysfunction of the sodium–potassium pump and calcium pump induces an imbalance in ion homeostasis and mitochondrial membrane damage, resulting in energy metabolism disorders [36]. Thus, to confirm the ameliorating effect of the extracts of *Lonicera japonica* on mitochondrial dysfunction, production of ROS, the MMP level, and ATP content in pulmonary tissues were assessed, and the extracts significantly inhibited pulmonary mitochondrial dysfunction (Figure 4). The extract of *Lonicera japonica* flower showed a protective effect against glutamate excitotoxicity by regulating Ca^2+^ and nitric oxide (NO) levels, enzymes related to the antioxidant system, cellular oxidation, and MMP production [40]. Moreover, the extract protected against mitochondrial damage from hydroxydopamine-induced cytotoxicity through the mitogen-activated protein kinase (MAPK)/phosphoinositide 3-kinase (PI3K)/protein kinase B (Akt) pathway [41]. It was reported that compounds of secoiridoids including secologanin, secologanic acid, and their metabolites, ameliorated mitochondrial dysfunction and decreased oxidative stress with their various physiological activities, such as a neuroprotective effect, anti-inflammatory response, and antioxidant activities [42]. In addition, 3,5-DCQA, a kind of DCQA, protected against mitochondrial damage from trimethyltin-induced cytotoxicity via the regulation of apoptosis [43]. The extract of *Lonicera japonica* containing secoiridoids and phenolic compounds with various physiological activities significantly suppressed the mitochondrial dysfunction induced by PM_2.5_ exposure. Therefore, it might be a substance that can help protect lung health from PM_2.5_. 

PM_2.5_ is associated with inflammatory cytokines and stimulates the overexpression of several transcription factor genes and inflammation-related cytokine genes in various tissues, resulting in extensive inflammatory damage [28]. Inflammation induced by PM_2.5_ increases the number of neutrophils in blood and eosinophils, T cells, and mast cells in bronchoalveolar lavage fluid [44]. Stimulated inflammatory response secretes cytokines and chemokines, including interleukin-2 (IL-2), interleukin-12 (IL-12), interferon gamma (IFNγ), and monocyte chemoattractant protein (MCP)-1, in nasal capacity and lung tissue [45]. These resulting increased cytokines can induce the migration of neutrophils, T cells, and eosinophils to the lungs and other tissues, where they can migrate to the lungs themselves and release more inflammatory cytokines and chemokines [46]. The increase in Th2 cells increases the production of IgE and IgG through the secretion of IL-4 and the stimulation of B cells, which causes chronic airway inflammation [45]. As a result, increased inflammatory mediators activate alveolar macrophages and neutrophils by activating MMPs and causing lung tissue damage [38]. It has been reported that this eventually damages lung tissue continuously and chronically through the interaction between inflammatory cells and cytokines. In addition, in a previous study, exposure to PM_2.5_ caused an inflammatory reaction by upregulating the protein expression of TNF-α, p-JNK, p-IκB-α, p-NF-κB, BAX, Caspase-1, COX-2, and IL-1β in pulmonary tissue, and TNF-α, TLR-4, TLR-2, p-JNK, BAX, and COX-2 in dermal tissue [47]. Thus, to assess the anti-inflammatory effect of the extracts of *Lonicera japonica*, protein levels of TLR-4, p-JNK, p-IκB-α, p-NF-κB, COX-2, iNOS, TNF-α, and IL-1β in pulmonary tissues were assessed, and the extracts significantly suppressed inflammation in serum and lung tissue (Figure 2, Figure 3, and Figure 6). In a previous study, 3,5-DCQA, a DCQA, suppressed LPS-induced microglial-activation-related inflammation via the MCP3/Janus tyrosine kinase 2 (JAK2)/signal transducer and activator of the transcription (STAT3) signal pathway, and LPS-treated inflammatory response by regulating the gene expression levels of iNOS, COX-2, and TNF-α [48,49]. The 4,5-DCQA presented anti-inflammatory effects via the NF-κB/MAPK pathway [50]. In addition, secoxyloganin and secologanic acid inhibited inflammation by regulating the gene expression of the adenosine A1 receptor (ADORA1), galectin-3, nucleotide-binding oligomerization domain 2 (NOD2), alpha-L-fucosidase 1 (FUCA1), and selectin P [51]. The administration of the extract of *Lonicera japonica* flower containing significant loganin content suppressed airway and lung inflammation by decreasing the TNF-α and IL-6 levels in bronchoalveolar lavage fluid (BALF) and the influx of neutrophils and total inflammatory cells in an LPS-induced BALB/c mice model [16]. Moreover, the inhalable microparticles of the flower of *Lonicera japonica* downregulated the levels of neutrophil, eosinophil, and basophil in peripheral blood in cigarette smoke and LPS-induced BALB/c mice [52]. In particular, the expression of COX-2 and iNOS, which were significantly increased in this study, increased lung permeability and neutrophil recruitment [53]. Through their expression increase, the expression of CXC chemokine, which attracts neutrophils, and CC chemokine, which attracts lymphocytes and monocytes to the lung, is increased [54]. In addition, the increased NO content by iNOS induces DNA strand breakage and base alteration, and increases the expression of ROS and RNS content by reacting with oxygen and superoxide anion [55]. Therefore, increased overexpression of COX-2 and iNOS can continuously promote lung tissue damage by stimulating a sustained inflammatory response. Based on these studies, the extract of the *Lonicera japonica* with various bioactive compounds, such as phenolic acids and secoiridoids, can significantly attenuate inflammatory reactions by regulating the various inflammatory pathways related to the NF-κB signal in serum and lung tissue.

PM_2.5_ not only causes an inflammatory reaction, but also promotes the generation of oxidative stress, which causes cell damage through a decrease in the antioxidant system [8]. Dysfunction of the antioxidant system due to PM_2.5_ exposure results in cell membrane damage in lung tissue and increases the intracellular Ca^2+^ level [56]. Through this process, apoptotic signals are stimulated by upregulating the expression of BAX, BCl-2 homologous antagonist killer (Bak), BCl-2-interacting mediator of cell death (Bim), and caspases related to apoptotic cascade [57]. Apoptosis caused by PM_2.5_ causes abnormal lung function, and stimulation of apoptosis due to continuous PM_2.5_ exposure causes pulmonary diseases such as pulmonary fibrosis and lung cancer [58]. Thus, to assess the protective effect of the extracts of *Lonicera japonica*, apoptotic protein levels of BCl-2, BAX, and Caspase-3 in pulmonary tissues were assessed, and the extracts significantly downregulated apoptosis in lung tissue (Figure 7). In a previous study, the polyphenols of *Lonicera japonica* suppressed cell apoptotic signals by decreasing the activation of cleaved poly-ADP-ribose polymerase (PARP), Caspase-3, Caspase-9, and BAX, and increasing the activation of BCl-2 and B-cell lymphoma-extra-large (Bcl-xL) in SH-SY5Y cells [41]. The 4,5-DCQA methyl ester isolated from *Lonicera japonica* attenuated H_2_O_2_-induced cytotoxicity by decreasing the BAX/BCl-2 and Bak levels and increasing the Kelch-like ECH-associated protein 1 (Keap1)/nuclear factor erythroid (Nrf) 2 level in HepG2 cells [59]. Chlorogenic acid protected against H_2_O_2_-indcued oxidative stress by regulating the protein expression of heme oxygenasw-1 (HO-1), Nrf1, and Akt in MC3T3-E1 cells [60]. In conclusion, the extract of *Lonicera japonica* containing phenolic acids significantly attenuated the apoptotic signals caused by PM_2.5_ inhalation.

TGF-β plays an important role in damage and repair signals in lung tissue [61]. Lung fibroblasts and myofibroblasts secrete TGF-β1 to protect tissues from external damage and promote phosphorylation of Smad2/3 with the activation of TGF-β1 receptors [62]. Activated Smad proteins cooperate with transcription factors to modulate various biological effects by regulating transcription according to the cellular state [63]. In addition, activated Smad signals stimulated subsignals, such as to levels of type I collagen (Col1), α-smooth muscle actin (Acta2/α-SMA), and fibroblast-specific protein 1 (Fsp1/S100A4), related to collagen accumulation and lung fibrosis [64]. Moreover, TGF-β activates the expression and secretion of MMP2 and MMP9 and downregulates the expression of inhibitors of MMPs [65]. Increased MMP activities stimulate the secretion of TGF-β1 and continuously activate the TGF-β1/Smad pathway [66]. Ultimately, this can continuously cause damage, fibrosis, and cancer of lung tissue. Therefore, the PM_2.5_-induced activation of TGF can stimulate Smad and MMP signaling, resulting in abnormal toxicity, including lung tissue damage and fibrosis, through various pathways. Thus, in this study, to evaluate the regulating effect of the extracts of *Lonicera japonica* in the TGF-β pathway, protein levels of TGF-β1, p-Smad-2, p-Smad-3, MMP-1, and MMP-2 in pulmonary tissues were assessed (Figure 8 and Figure 9). Similar to these results, the extract of *Lonicera japonica* decreased activities of MMP-2 and MMP-9 against ovalbumin-induced allergic asthma [18]. Chlorogenic acid prevented bleomycin-induced pulmonary fibrosis by suppressing the protein expression of collagen I, α-SMA, and glucose-regulated protein 78 (GRP78) in a dose-dependent manner [67]. Quercetin protected elastase/LPS-exposed COPD by decreasing the expression of cytokines, mucin 5AC (muc5ac), MMP9, and MMP12 in lung tissue [68]. In particular, this study presented that the protein expression level of MMP-2 was significantly upregulated relative to the expression of MMP-1 (Figure 9). The role of MMP-1, a gelatinase, is related to emphysematous changes rather than fibrotic changes [69]. On the other hand, MMP-2, a fibrillar collagenase related to matrix protein homeostasis, is a factor that directly affects fibrosis along with MMP-8 and MMP-13 [70]. The excessive increase of MMP-2 stimulates relatively more pulmonary fibrosis induced by PM_2.5_ than MMP-1. In conclusion, the extract of *Lonicera japonica* with various bioactive compounds, such as phenolic compounds, significantly protected against the pulmonary fibrosis induced by PM_2.5_ exposure via the TGF-β/Smad/MMP pathway. 

## 5. Conclusions

**Figure 10:**
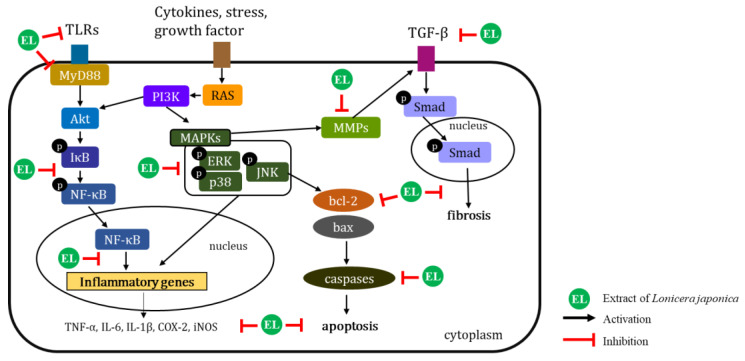
A schematic illustration shows the protective effect of extract of *Lonicera japonica* in particulate-matter (PM)_2.5_-induced pulmonary dysfunction in BALB/c mice via regulation of the TGF-β/Smad/MMP signaling pathway.

In summary, the extracts of *Lonicera japonica* containing various phenolic compounds presented a significant pulmonary protective effect against PM_2.5_-induced cellular cytotoxicity in A549 cells. The extracts suppressed inflammatory T cells and immunoglobulin levels in chronic PM_2.5_-induced BALB/c mice. The extracts improved the antioxidant system and mitochondrial dysfunction in lung tissues. Furthermore, the extracts suppressed inflammatory responses by regulating TLR-4, p-JNK, p-IκB-α, p-NF-κB, COX-2, iNOS, TNF-α, and IL-1β. In addition, the extracts restored apoptotic signaling by regulating the protein expression levels of BCl-2, BAX, and Caspase-3, and the fibrosis pathway by regulating the protein expression levels of TGF-β1, p-Smad-2, p-Smad-3, MMP-1, and MMP-2 in lung tissues. In conclusion, based on this study, it is suggested that the extracts of *Lonicera japonica* might be used as a material for functional foods or alternative medicines to improve lung health (Figure 10).

## Figures and Tables

**Table 1:** List of primary antibody information used in this study.

Antibody	Catalog	Concentration	Manufacturer
TLR-4	sc-52962	1:1000	Santa Cruz Biotech (Dallas, TX, USA)
p-JNK	sc-6254	1:1000	Santa Cruz Biotech (Dallas, TX, USA)
p-NF-κB	3033	1:1000	Cell Signaling Tech (Danvers, MA, USA)
p-IκB-α	#sc-8404	1:1000	Santa Cruz Biotech (Dallas, TX, USA)
COX-2	sc-376861	1:1000	Santa Cruz Biotech (Dallas, TX, USA)
iNOS	sc-7271	1:1000	Santa Cruz Biotech (Dallas, TX, USA)
TNF-α	sc-393887	1:1000	Santa Cruz Biotech (Dallas, TX, USA)
IL-1β	sc-4592	1:1000	Santa Cruz Biotech (Dallas, TX, USA)
BCl-2	sc-509	1:1000	Santa Cruz Biotech (Dallas, TX, USA)
BAX	sc-7480	1:1000	Santa Cruz Biotech (Dallas, TX, USA)
Caspase-3	CSB-PA05689A0Rb	1:1000	Cusabio (Wuhan, China)
TGF-β1	sc-130348	1:1000	Santa Cruz Biotech (Dallas, TX, USA)
p-Smad-2	#3108	1:1000	Cell Signaling Tech (Danvers, MA, USA)
p-Smad-3	sc-517575	1:1000	Santa Cruz Biotech (Dallas, TX, USA)
MMP-1	sc-21731	1:1000	Santa Cruz Biotech (Dallas, TX, USA)
MMP-2	sc-13595	1:1000	Santa Cruz Biotech (Dallas, TX, USA)
β-actin	66009-1-Ig	1:1000	Proteintech (Rosemont, IL, USA)

**Table 2:** Identification of main compounds of *Lonicera japonica*.

No.	Retention Time (min)	Parent Ion (*m/z*)	MS^E^ Fragment (*m/z*)	Compound
**1**	2.92	391	149,167	Shanzhiside
**2**	3.00	389	165,183,345	Secologanoside
**3**	3.36	375	99,116,151,195,341	Loganic acid
**4**	3.41	353	191	Chlorogenic acid
**5**	3.49	373	149,167,179,193	Secologanic acid
**6**	3.78	403	121,165,223,371	Secoxyloganin
**7**	3.83	433	271,300,301	Quercetin pentoside
**8**	4.11	515	133,135,179,191,353	3,4-O-DCQA *
**9**	4.14	515	116,179,191,353	3,5-O-DCQA
**10**	4.19	515	135,173,179,191,353	4,5-O-DCQA
**11**	4.26	515	161,173,191,353	1,4-O-DCQA

* DCQA: Dicaffeoyl quinic acid.

## Data Availability

The data underlying this article are shared upon reasonable request to the corresponding author.

## References

[1] Ling S.H., van Eeden S.F. (2009). Particulate matter air pollution exposure: Role in the development and exacerbation of chronic obstructive pulmonary disease. Int. J. Chronic Obstr. Pulm. Dis..

[2] Kermani M., Rahmatinia T., Oskoei V., Norzaee S., Shahsavani A., Farzadkia M., Kazemi M.H. (2021). Potential cytotoxicity of trace elements and polycyclic aromatic hydrocarbons bounded to particulate matter: A review on in vitro studies on human lung epithelial cells. Environ. Sci. Pollut. Res..

[3] Ma Q.Y., Huang D.Y., Zhang H.J., Wang S., Chen X.F. (2017). Exposure to particulate matter 2.5 (PM2.5) induced macrophage-dependent inflammation, characterized by increased Th1/Th17 cytokine secretion and cytotoxicity. Int. Immunopharmacol..

[4] Mukherjee A., Agrawal S.B., Agrawal M. (2020). Responses of tropical tree species to urban air pollutants: ROS/RNS formation and scavenging. Sci. Total Environ..

[5] Della Guardia L., Shin A.C. (2022). The role of adipose tissue dysfunction in PM_2.5_-induced vascular pathology. Am. J. Physiol. Heart Circ. Physiol..

[6] Zhu R.X., Nie X.H., Chen Y.H., Chen J., Wu S.W., Zhao L.H. (2020). Relationship between particulate matter (PM_2.5_) and hospitalizations and mortality of chronic obstructive pulmonary disease patients: A meta-analysis. Am. J. Med. Sci..

[7] Atkinson R.W., Carey I.M., Kent A.J., Van Staa T.P., Anderson H.R., Cook D.G. (2015). Long-term exposure to outdoor air pollution and the incidence of chronic obstructive pulmonary disease in a national English cohort. Occup. Environ. Med..

[8] Wang W., Kang P.M. (2020). Oxidative stress and antioxidant treatments in cardiovascular diseases. Antioxidants.

[9] Wyzga R.E., Rohr A.C. (2015). Long-term particulate matter exposure: Attributing health effects to individual PM components. J. Air Waste Manag. Assoc..

[10] Li Y., Xie L., Liu K., Li X., Xie F. (2023). Bioactive components and beneficial bioactivities of flowers, stems, leaves of *Lonicera japonica* Thunberg: A review. Biochem. Syst. Ecol..

[11] Rupasinghe H.V., Arumuggam N., Amararathna M., De Silva A.B.K.H. (2018). The potential health benefits of haskap (*Lonicera caerulea* L.): Role of cyanidin-3-O-glucoside. J. Funct. Foods.

[12] Xiong J., Li S., Wang W., Hong Y., Tang K., Luo Q. (2013). Screening and identification of the antibacterial bioactive compounds from *Lonicera japonica* Thunb. leaves. Food Chem..

[13] Tzeng T.F., Tzeng Y.C., Cheng Y.J., Liou S.S., Liu I.M. (2015). The ethanol extract from *Lonicera japonica* Thunb. regresses nonalcoholic steatohepatitis in a methionine-and choline-deficient diet-fed animal model. Nutrients.

[14] Wang J., Liu P., Huang X., Wu X. (2021). Validation of the protective effects of *Lonicera japonica* polysaccharide on lipopolysaccharide-induced learning and memory impairments via regulation of autophagy based on network pharmacology. Ann. Palliat. Med..

[15] Zhou X., Lu Q., Kang X., Tian G., Ming D., Yang J. (2021). Protective role of a new polysaccharide extracted from *Lonicera japonica* Thunb in mice with ulcerative colitis induced by dextran sulphate sodium. BioMed Res. Int..

[16] Lee H., Lee D., Kim Y., Lee G., Kim S.J., Jung S., Jung H., Bae H. (2011). Lipopolysaccharide induced lung inflammation is inhibited by *Lonicera japonica*. Mol. Cell. Toxicol..

[17] Lee H., Lee G., Yoon M.S., Hong M., Shin M., Bae H. (2011). Effect of fermented *Lonicera japonica* on LPS-induced acute lung inflammation. Orient. Pharm. Exp. Med..

[18] Hong S.-H., Kwon J.-T., Shin J.-Y., Kim J.-E., Minai-Tehrani A., Yu K.-N., Lee S., Park S.-J., Chang S.-H., Jiang H.-L. (2013). Therapeutic effect of Broussonetia papyrifera and Lonicera japonica in ovalbumin-induced murine asthma model. Nat. Prod. Commun..

[19] Jin L., Deng L., Bartlett M., Ren Y., Lu J., Chen Q., Pan Y., Wang H., Guo X., Liu C. (2022). A novel herbal extract blend product prevents particulate matters-induced inflammation by improving gut microbiota and maintaining the integrity of the intestinal barrier. Nutrients.

[20] Kim H.Y., Yoon J.J., Kim D.S., Kang D.G., Lee H.S. (2021). Yg-1 extract improves acute pulmonary inflammation by inducing bronchodilation and inhibiting inflammatory cytokines. Nutrients.

[21] Kim J.M., Park S.K., Kang J.Y., Park S.B., Yoo S.K., Han H.J., Kim C.W., Lee U., Kim S.H., Heo H.J. (2018). Ethyl acetate fraction from persimmon (*Diospyros kaki*) ameliorates cerebral neuronal loss and cognitive deficit via the JNK/Akt pathway in TMT-induced mice. Int. J. Mol. Sci..

[22] Kim J.M., Park S.K., Kang J.Y., Park S.B., Yoo S.K., Han H.J., Cho K.H., Kim J.C., Heo H.J. (2019). Green tea seed oil suppressed Aβ_1–42_-induced behavioral and cognitive deficit via the Aβ-related Akt pathway. Int. J. Mol. Sci..

[23] Kim J.M., Lee U., Kang J.Y., Park S.K., Kim J.C., Heo H.J. (2020). Matcha improves metabolic imbalance-induced cognitive dysfunction. Oxidative Med. Cell. Longev..

[24] Kim J.M., Park S.K., Guo T.J., Kang J.Y., Ha J.S., Lee D.S., Lee U., Heo H.J. (2016). Anti-amnesic effect of *Dendropanax morbifera* via JNK signaling pathway on cognitive dysfunction in high-fat diet-induced diabetic mice. Behav. Brain Res..

[25] Zhang Y.D., Huang X., Zhao F.L., Tang Y.L., Yin L. (2015). Study on the chemical markers of Caulis *Lonicerae japonicae* for quality control by HPLC-QTOF/MS/MS and chromatographic fingerprints combined with chemometrics methods. Anal. Methods.

[26] Jiang M., Han Y.Q., Zhou M.G., Zhao H.Z., Xiao X., Hou Y.Y., Luo G.A. (2014). The screening research of anti-inflammatory bioactive markers from different flowering phases of Flos *Lonicerae Japonicae*. PLoS ONE.

[27] Chang R., Chen W., Zhou T. (2019). A sensitive LC-MS/MS method for simultaneous assay of three iridiods in *Gardenia jasminoides* Ellis. J. Pharm. Pract..

[28] Xing Y.F., Xu Y.H., Shi M.H., Lian Y.X. (2016). The impact of PM2.5 on the human respiratory system. J. Thorac. Dis..

[29] Xiong R., Jiang W., Li N., Liu B., He R., Wang B., Geng Q. (2021). PM2.5-induced lung injury is attenuated in macrophage-specific NLRP3 deficient mice. Ecotoxicol. Environ. Safe.

[30] Zhu X., Ji X., Shou Y., Huang Y., Hu Y., Wang H. (2020). Recent advances in understanding the mechanisms of PM2. 5-mediated neurodegenerative diseases. Toxicol. Lett..

[31] Zhao X., Wang D., Qin L., Yang X., Gao C. (2018). Comparative investigation for hypoglycemic effects of polysaccharides from four substitutes of *Lonicera japonica* in Chinese medicine. Int. J. Biol. Macromol..

[32] Jun C.H., Song C.H. (2021). Inhibitory effect of *Lonicera japonica* Thunb. flower buds against glutamate-induced cytotoxicity in HT22 hippocampal neurons. Korean J. Acupunct..

[33] Bang B.W., Park D., Kwon K.S., Lee D.H., Jang M.J., Park S.K., Kim J.Y. (2019). BST-104, a water extract of *Lonicera japonica*, has a gastroprotective effect via antioxidant and anti-inflammatory activities. J. Med. Food.

[34] Zhang T., Chen S., Chen L., Zhang L., Meng F., Sha S., Ai C., Tai J. (2019). Chlorogenic acid ameliorates lead-induced renal damage in mice. Biol. Trace Elem. Res..

[35] Bardestani A., Ebrahimpour S., Esmaeili A., Esmaeili A. (2021). Quercetin attenuates neurotoxicity induced by iron oxide nanoparticles. J. Nanobiotechnol..

[36] Guo Z., Hong Z., Dong W., Deng C., Zhao R., Xu J., Zhuang G., Zhang R. (2017). PM_2.5_-induced oxidative stress and mitochondrial damage in the nasal mucosa of rats. Int. J. Environ. Res. Public Health.

[37] Ning X., Ji X., Li G., Sang N. (2019). Ambient PM_2.5_ causes lung injuries and coupled energy metabolic disorder. Ecotoxicol. Environ. Safe.

[38] Zeng X., Liu D., Wu W., Huo X. (2022). PM_2.5_ exposure inducing ATP alteration links with NLRP3 inflammasome activation. Environ. Sci. Pollut. Res..

[39] Li R., Kou X., Geng H., Xie J., Yang Z., Zhang Y., Dong C. (2015). Effect of ambient PM_2.5_ on lung mitochondrial damage and fusion/fission gene expression in rats. Chem. Res. Toxicol..

[40] Ma C.J., Weon J.B., Lee B., Ahn J.H., Yang H.J., Yun B.-R., Lee H.Y. (2011). Neuroprotective activity of the methanolic extract of *Lonicera japonica* in glutamate-injured primary rat cortical cells. Pharmacogn. Mag..

[41] Kwon S.H., Hong S.I., Jung Y.H., Kim M.J., Kim S.Y., Kim H.C., Lee S.Y., Jang C.G. (2012). *Lonicera japonica* THUNB. protects 6-hydroxydopamine-induced neurotoxicity by inhibiting activation of MAPKs, PI3K/Akt, and NF-κB in SH-SY5Y cells. Food Chem. Toxicol..

[42] Peng Z., He J., Cheng Y., Xu J., Zhang W. (2023). Biologically active secoiridoids: A comprehensive update. Med. Res. Rev..

[43] Kang J.Y., Park S.K., Guo T.J., Ha J.S., Lee D.S., Kim J.M., Lee U., Kim D.O., Heo H.J. (2016). Reversal of trimethyltin-induced learning and memory deficits by 3, 5-dicaffeoylquinic acid. Oxidative Med. Cell. Longev..

[44] Sigaud S., Goldsmith C.A.W., Zhou H., Yang Z., Fedulov A., Imrich A., Kobzik L. (2007). Air pollution particles diminish bacterial clearance in the primed lungs of mice. Toxicol. Appl. Pharmacol..

[45] Yang J., Chen Y., Yu Z., Ding H., Ma Z. (2019). The influence of PM2.5 on lung injury and cytokines in mice. Exp. Ther. Med..

[46] He M., Ichinose T., Yoshida S., Nishikawa M., Mori I., Yanagisawa R., Takano H., Inoue K., Sun G., Shibamoto T. (2010). Urban particulate matter in Beijing, China, enhances allergen-induced murine lung eosinophilia. Inhal. Toxicol..

[47] Kim J.M., Kang J.Y., Park S.K., Moon J.H., Kim M.J., Lee H.L., Jeong H.R., Kim J.C., Heo H.J. (2021). Powdered green tea (matcha) attenuates the cognitive dysfunction via the regulation of systemic inflammation in chronic PM_2.5_-exposed BALB/c mice. Antioxidants.

[48] Park J., Kim Y., Lee C., Kim Y.T. (2022). 3,5-Dicaffeoylquinic acid attenuates microglial activation-mediated inflammatory pain by enhancing autophagy through the suppression of MCP3/JAK2/STAT3 signaling. Biomed. Pharmacother..

[49] Hong S., Joo T., Jhoo J.W. (2015). Antioxidant and anti-inflammatory activities of 3,5-dicaffeoylquinic acid isolated from *Ligularia fischeri* leaves. Food Sci. Biotechnol..

[50] Jang G., Lee S., Hong J., Park B., Kim D., Kim C. (2021). Anti-inflammatory effect of 4,5-dicaffeoylquinic acid on RAW264.7 cells and a rat model of inflammation. Nutrients.

[51] Guo X., Yu X., Zheng B., Zhang L., Zhang F., Zhang Y., Li J., Pu G., Zhang L., Wu H. (2021). Network pharmacology-based identification of potential targets of *Lonicerae japonicae* flos acting on anti-inflammatory effects. BioMed Res. Int..

[52] Park Y.C., Jin M., Kim S.H., Kim M.H., Namgung U., Yeo Y. (2014). Effects of inhalable microparticle of flower of *Lonicera japonica* in a mouse model of COPD. J. Ethnopharmacol..

[53] Speyer C.L., Neff T.A., Warner R.L., Guo R.F., Sarma J.V., Riedemann N.C., Murphy M.E., Murphy H.S., Ward P.A. (2003). Regulatory effects of iNOS on acute lung inflammatory responses in mice. Am. J. Pathol..

[54] Olson T.S., Ley K. (2002). Chemokines and chemokine receptors in leukocyte trafficking. Am. J. Physiol. Regul. Integr. Comp. Physiol..

[55] Weinberger B., Laskin D.L., Heck D.E., Laskin J.D. (2001). The toxicology of inhaled nitric oxide. Toxicol. Sci..

[56] Zheng L., Wang Y., Zhang Y., Fu Y., Yang Z., Fan Y., Sun Z., Zhao M., Zhu L., Dai B. (2021). EGFR inhibitors regulate Ca^2+^ concentration and apoptosis after PM_2.5_ exposure based on a lung-mimic microfluidic system. Sci. Total Environ..

[57] Foo J., Bellot G., Pervaiz S., Alonso S. (2022). Mitochondria-mediated oxidative stress during viral infection. Trends Microbiol..

[58] Liu S., Zhang W., Zhang F., Roepstorff P., Yang F., Lu Z., Ding W. (2019). TMT-based quantitative proteomics analysis reveals airborne PM_2.5_-induced pulmonary fibrosis. Int. J. Environ. Res. Public Health.

[59] Xiao L., Liang S., Ge L., Wan H., Wu W., Fei J., Wu S., Zhou B., Zeng X. (2020). 4,5-di-O-caffeoylquinic acid methyl ester isolated from *Lonicera japonica* Thunb. targets the Keap1/Nrf2 pathway to attenuate H_2_O_2_-induced liver oxidative damage in HepG2 cells. Phytomedicine.

[60] Han D., Chen W., Gu X., Shan R., Zou J., Liu G., Shahid M., Gao J., Han B. (2017). Cytoprotective effect of chlorogenic acid against hydrogen peroxide-induced oxidative stress in MC3T3-E1 cells through PI3K/Akt-mediated Nrf2/HO-1 signaling pathway. Oncotarget.

[61] Saito A., Horie M., Nagase T. (2018). TGF-β signaling in lung health and disease. Int. J. Mol. Sci..

[62] Wójcik K.A., Skoda M., Koczurkiewicz P., Sanak M., Czyż J., Michalik M. (2013). Apigenin inhibits TGF-β1 induced fibroblast-to-myofibroblast transition in human lung fibroblast populations. Pharmacol. Rep..

[63] Wang Z.J., Yu H., Hao J.J., Peng Y., Yin T.T., Qiu Y.N. (2021). PM_2.5_ promotes Drp1-mediated mitophagy to induce hepatic stellate cell activation and hepatic fibrosis via regulating miR-411. Exp. Cell Res..

[64] Ma K., Li C., Xu J., Ren F., Xu X., Liu C., Niu B., Li F. (2020). LncRNA Gm16410 regulates PM_2.5_-induced lung endothelial-mesenchymal transition via the TGF-β1/Smad3/p-Smad3 pathway. Ecotoxicol. Environ. Saf..

[65] Liu S., Chen S., Zeng J. (2018). TGF-β signaling: A complex role in tumorigenesis. Mol. Med. Rep..

[66] Wang Y., Zhang Y., Li Y., Kou X., Xue Z. (2022). Mechanisms of biochanin a alleviating PM2.5 organic extracts-induced EMT of A549 cells through the PI3K/Akt pathway. J. Nat. Prod..

[67] Wang Y.C., Dong J., Nie J., Zhu J.X., Wang H., Chen Q., Chen J.Y., Xia J.M., Shuai W. (2017). Amelioration of bleomycin-induced pulmonary fibrosis by chlorogenic acid through endoplasmic reticulum stress inhibition. Apoptosis.

[68] Ganesan S., Faris A.N., Comstock A.T., Chattoraj S.S., Chattoraj A., Burgess J.R., Curtis J.L., Martinez F.J., Zick S., Hershenson M.B. (2010). Quercetin prevents progression of disease in elastase/LPS-exposed mice by negatively regulating MMP expression. Respir. Res..

[69] Ouchi H., Fujita M., Ikegame S., Ye Q., Inoshima I., Harada E., Kuwano K., Nakanishi Y. (2008). The role of collagenases in experimental pulmonary fibrosis. Pulm. Pharmacol. Ther..

[70] Chuliá-Peris L., Carreres-Rey C., Gabasa M., Alcaraz J., Carretero J., Pereda J. (2022). Matrix metalloproteinases and their inhibitors in pulmonary fibrosis: EMMPRIN/CD147 comes into play. Int. J. Mol. Sci..

